# Rb interactome data and its modulations during cell cycle progression in HEK 293 cells

**DOI:** 10.1016/j.dib.2018.01.045

**Published:** 2018-01-31

**Authors:** Shweta Duggal, Noor Jailkhani, Mukul Kumar Midha, Kanury V.S. Rao, Ajay Kumar

**Affiliations:** a International Centre for Genetic Engineering and Biotechnology (ICGEB), Aruna Asif Ali Marg, New Delhi 110067, India; b David H. Koch Institute for Integrative Cancer Research, Department of Biology, Massachusetts Institute of Technology, Cambridge, MA 02139, United States; c Drug Discovery Research Center (DDRC), Translational Health Science and Technology Institute (THSTI), NCR Biotech Science Cluster, 3rd Milestone, Faridabad-Gurgaon Expressway, Faridabad 121001, Haryana, India

**Keywords:** Rb, Cell cycle, Interactome, SILAC, AP-MS, HEK 293 cells

## Abstract

The Rb protein is a tumor suppressor protein that regulates the key G1S checkpoint consequently blocking the progression of cell cycle into S-phase. Despite its pertinent role in cell cycle regulation, comprehensive information on its interacting partners across cell cycle progression is lacking. Here, we aim to submit a comprehensive set of Rb interactors as the cell progresses from G0 through G1 and S into G2 phase in HEK 293 cell line. Affinity purification of HA-tagged Rb protein along with its interactors was analyzed by mass spectrometry (AP-MS). SILAC labeling enabled differentiation of Rb interactors in different cell cycle stages as well as their quantification - G0 cells were labeled with light labels of lysine and arginine (K0R0), cells in G1S transition were labeled with heavy labels (K8R10) while the G2 cells were labeled with medium labels (K6R6). LC-MS/MS analysis resulted in 6 wiff files which were submitted to protein pilot software for peptide identification and quantification. Here we submit the dataset which clearly captures the changing interacting partners of the Rb protein as the cell cycle progressed from G0 through G1S checkpoint into G2 phase. Data is publicly available via ProteomeXchange with identifier PXD007708.

**Specifications Table**

**Table:** 

Subject area	*Biology*
More specific subject area	*Proteomics*
Type of data	*Mass Spectrometry raw files, Figure*
How data was acquired	*Mass Spectroscopy, AB Sciex 5600 Triple TOF*
Data format	*Raw and analyzed*
Experimental factors	*Rb1 expressing cell line arrested at different cell cycle stages: G0, G1S transition and G2 phase*
Experimental features	*SILAC labeling, LysC - Trypsin - Chymotrypsin Digestion, peptide separation through nano-flow liquid chromatography on a nanoflex system coupled to a triple TOF 5600 mass spectrometer (AB Sciex)*
Data source location	*New Delhi, India*
Data accessibility	*Data is within this article and available in a Public repository via ProteomeXchange with identifier ProteomeXchange:*PXD007708

**Value of the data**

• Our dataset highlights changing Rb protein interactors as the cell cycle advances from G0 through G1S transition and G2 phase in HEK 293 cell line.
• Our data corroborates some of the reported interactors of Rb protein as well as suggests new proteins as its interactors.
• Association dynamics of the identified protein partners of Rb protein could form the basis for future research to understand cell cycle regulation.

## 1 Data

**Fig. 1:**
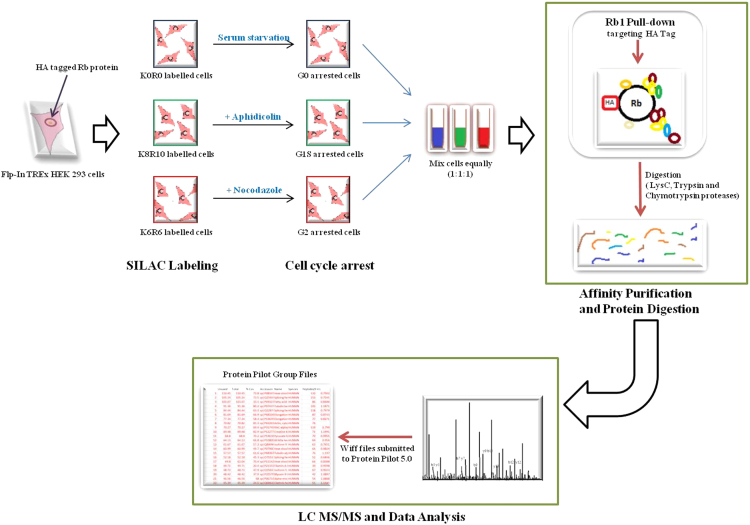
Schematic overview showing the experimental workflow.

In this study, we attempt to delineate cell cycle-dependent protein interactors of Rb protein. HA-tagged Rb1 gene was expressed in HEK 293 cells. Rb protein and its interacting partners were affinity-purified by targeting the HA tag. SILAC (Stable Isotope Labeling in Cell Culture) labeling strategy was used to capture cell cycle phase-specific interacting protein partners of Rb protein. Cell cycle was arrested at G0, G1S transition and G2 phase in the Rb1 expressing HEK 293 cells using serum starvation, aphidicolin, and nocodazole, respectively. Prior to arresting cells in specific cell cycle phases, cells to be arrested in G0 phase were labeled with light SILAC labels (L0R0), those to be arrested in G1S transition were labeled with heavy SILAC labels (L8R10) and G2 cells were labeled with medium SILAC labels (L6R6). SILAC labeling was instrumental in differentiating between cell cycle phase specific interactomes. Three biological replicates of each sample representing a specific cell cycle phase were affinity purified and analyzed separately using AB Sciex 5600 triple TOF mass spectrometer. The results were obtained as 6 RAW file pairs (wiff and corresponding wiff.scan files). Wiff files were submitted to protein pilot software version 5.0 and resulted in 18 protein pilot group files (6 group files per biological replicate). The experimental scheme is shown in Fig. 1. Data is publicly available via ProteomeXchange with identifier ProteomeXchange: PXD007708.

## 2 Experimental design, materials and methods

### 2.1 Generation of Rb expressing cell line

To generate tetracycline (Tet) inducible Rb1 expression vector, the pENTR221 vector including Rb1 ORF (purchased from GE Dharmacon) was subjected to LR recombination along with modified destination vector (pcDNA/FRT/TO). The destination vector was modified to include an N-terminal HA tag for selective separation of Rb protein and was a generous gift from Dr. Matthias Gstaiger (Institute of Molecular Systems Biology, ETH Zurich, Zurich, Switzerland).

Flp-In T-REx HEK 293 cells (Invitrogen), stably expressing the Tet-repressor, were cultured in 10% DMEM medium containing 100 µg/ml zeocin and 15 µg/ml blasticidin. The Rb1 expression vector was co-transfected with the pOG44 vector in these cells using Xtremegene 9 transfection reagent (Roche), as per manufacturer's instructions. Two days later, the transfected cells were transferred to hygromycin selection media (50 µg/ml) with 10% Tet screened FBS. The selection was continued over 2 weeks with media change twice a week. Optimal Tet dosage, required to induce Rb expression, was determined from the literature to be 1 µg/ml [1]. Tet (1 µg/ml) was supplemented in media of Rb expressing cells every 24 h to maintain Rb expression.

### 2.2 SILAC labeling and affinity purification

Rb expressing HEK 293 cells were cultured in SILAC media containing “light” (K0R0), “medium” (K6R6) or “heavy” (K8R10) isotopes of lysine (K) and arginine (R) for at least 5 cell doublings to allow complete label incorporation [2]. At 70% cell confluency, Tet was supplemented to K0R0, K6R6 and K8R10 labeled cell culture media. Twenty-four hours after Tet induction, cell cycle arrest was facilitated in G0 phase by overnight serum starvation [3]; G1/S transition in presence of 5 µg/ml aphidicolin by 16–18 h incubation [4]; and G2 phase in presence of 400 ng/µl nocodazole [5] by 16–18 h incubation. The arrested cells were trypsinized, counted by trypan blue staining method and pelleted by centrifugation at 1500 rpm for 10 min. Multiple pellets were generated for cells arrested in each cell cycle phase and stored at −80 °C, until used.

An equal number of Rb expressing cells representing G0, G1/S transition and G2 phase of the cell cycle were pooled and lysed for 1 h on ice in IP buffer (150 mM NaCl; 50 mM Tris–HCl pH7.5; 1% NP-40; 1× protease inhibitor cocktail and 1 mM PMSF) [6]. Cell debris was removed following centrifugal separation at 10,000 rpm for 15 min at 4 °C. Rb protein and its interacting partners were selectively purified by targeting the HA tag as instructed in the kit manual (Invitrogen). Briefly, cleared cell lysates were kept for incubation with 50 µl of pre-washed HA agarose beads for 2 h at 4 °C on a rotary shaker. After 3 quick washes with ten bead volumes of TBS-T buffer, Rb protein complexes were eluted thrice with 50 µl HA peptide (250 µg/ml) per elution step. Three such affinity purifications were performed as biological replicates and the eluates for each replicate set were pooled, lyophilized and saved at −80 °C, until further processing.

### 2.3 Protein digestion and sample preparation for LC-MS/MS

Each biological replicate was processed separately. Lyophilized eluates were re-suspended in 40 µl of 100 mM ammonium bicarbonate, vortexed well and supplemented with 2 µl of 2% SDS (denaturant buffer). Protein samples were reduced at 60 °C with 4 µl of 50 mM TCEP [Tris-(2-carboxyethyl) phosphine] for 1 h. Reduced cysteine residues were blocked with 2 µl of 200 mM MMTS (methyl methanethiosulfonate) by incubating for 10 min at room temperature. Digestion was initiated by adding 5 µl of 0.1 µg/µl endo-proteinase LysC and kept for incubation for 4 h in a 37 °C water bath. After a short spin, 0.5 µl of trypsin (1 µg/µl) and 0.5 µl of chymotrypsin (1 µg/µl) was supplemented and continued incubation for another 12–16 h at 37 °C. A drop of formic acid was used to terminate protein digestion.

Acidified samples were lyophilized and peptides were purified using monospin C-18 columns (Waters). Pre-conditioned C-18 columns were equilibrated with 3% acetonitrile (ACN) in 0.1% formic acid (FA). Lyophilized peptide samples were dissolved in 3% ACN in 0.1% FA and loaded onto C-18 columns and allowed to bind for 10 min. The samples were passed twice through the columns to ensure complete binding. After 10 stringent washes with 3% ACN in 0.1% FA, the digested peptides were eluted first in 40% ACN followed by two elutions in 60% ACN and one elution with 90% ACN. Finally, the four eluates were pooled and lyophilized.

In an effort to remove any remaining salt, the eluted peptides were re-dissolved in 500 µl of 5 mM ammonium formate (pH 2.5) in 30% ACN and gently vortexed to mix. The cation exchange cartridge was fixed and conditioned before loading the sample. Samples were loaded onto the cartridge followed by three wash steps with 1 ml of 5 mM ammonium formate. The peptides were re-eluted twice with 400 µl of 500 mM ammonium formate (pH 2.5) in 30% ACN; pooled and lyophilized.

### 2.4 LC-MS/MS analysis

All samples were analyzed by nano-flow liquid chromatography on a nano flex system (Eksigent Technologies, AB Sciex) coupled to a triple TOF 5600 mass spectrometer (AB Sciex; Concord, Canada). Each biological replicate was injected twice as technical replicates.

The system was operated using a binary phase gradient, with solvent A (2% ACN in 0.1% FA) and solvent B (98% ACN in 0.1% FA). For optimal sample delivery reproducibility, auto-sampler was operated in full injection mode overfilling the 1 µl loop with 3 µl of the sample. The peptides were trapped onto cHiPLC trap, 3 µm, Chrom XP C18CL, 120 Å, 0.5 mm×200 µm (Eksigent) and separated on a cHiPLC column, 3 µm, Chrom XP C18CL, 120 Å, 15 cm×75 µm (Eksigent) at 300 nl/min flow rate, using linear gradient: 5–60% B in 80 min, 60–90% for 2 min. The column was regenerated by washing with 90% solvent B for 6 min and re-equilibrated with 5% solvent B for 22 min.

The mass spectrometer was coupled to a Nano Spray Ion Source (AB Sciex), controlled by Analyst software (v.1.6). The ion source was equipped with a 10 μm SilicaTip electrospray PicoTip emitter (AB Sciex) and the eluted peptides were monitored by following ion source parameters - IHT=130°, ISVF=2100 v, GS1=20, curtain gas=25. The mass spectrometer was operated in information dependent acquisition (IDA) top 10 mode and high sensitivity mode with 500 and 200 ms accumulation time for the MS1 and MS2 scans respectively, and 12 s dynamic exclusion, resulting in a total duty cycle of ~2.55 s. Mass spectrometer analysis was performed using TOF-MS1 and MS2 survey scans ranging from 350 to 1250 and 140 to 1600 m/z, respectively. The rolling collision energy was automatically controlled by IDA rolling collision energy parameter script. Selection criteria for the parent ion to be fragmented included intensity - where ions had to be greater than 150 cps, mass tolerance of 50 mDa, with a charge state of +2 to +5. An auto-calibration of spectra was done after the acquisition of every 2 samples using dynamic LC-MS and MS/MS acquisitions of 25 fmol β-galactosidase.

### 2.5 Data processing and analysis

MS-MS acquisition of Rb and its interacting partners was mediated in G0 phase, G1/S transition and G2 phase of the cell cycle. SILAC labeled cells representing each phase were pooled in three separate sets and probed as biological replicates. Acquisition of each biological replicate resulted in 2 wiff and 2 wiff.scan files, representing its technical replicates. Wiff files were again pooled for each biological replicate before searching against UniProtKB/SwissProt Human database (release July 2017) using protein pilot software, version 5.0 (revision no. 4769). The reference database consisted of 20,218 protein entries in the specified release. Protein pilot search was based on the paragon algorithm, a part of default statistics of the software. The following settings were used for paragon searches - (a) Species as Homo sapiens, (b) Lys C + Trypsin and Chymotrypsin as enzyme categories for different runs, (c) Maximum missed cleavages=2, (d) Fixed modifications as SILAC labels - K6R6 and K8R10; cysteine alkylation by methyl methanethiosulphonate (MMTS), (e) Variable modifications as oxidation at methionine, which is a default option in protein pilot, (f) Identification, SILAC (Lys+6, Arg+6) and SILAC (Lys+8, Arg+10) as sample types, and (g) “Search Effort” parameter “Thorough ID”, which gives us a broad search of various protein modifications, (h) Mass tolerance for precursor and fragment ions were 0.05 and 0.1 Da, respectively. The following parameters were used for identification and quantification of differentially expressed proteins - (a) Auto Bias correction for heavy to light ratio. (b) The threshold of 1% accepted Global False discovery rate from fit (G-FDR-fit) proteins; (c) Minimum protein confidence threshold cutoff of 95%; (d) At least one peptide with 95% confidence for the relative expression.

For each biological replicate, six protein pilot files were generated: files representing 3 SILAC labels (light, medium and heavy) with two enzyme categories used for digestion (LysC + Trypsin and Chymotrypsin) were created individually. Here, the light label was represented as 0/0 (G0 stage), medium as 6/6 (G2 stage) and heavy as 8/10 (G1/S transition).

The mass spectrometry proteomics data have been deposited to the ProteomeXchange Consortium via the PRIDE [7] partner repository with the dataset identifier PXD007708.
